# A case report of tumor lysis syndrome after stage-one ALPPS

**DOI:** 10.1097/MD.0000000000029040

**Published:** 2022-03-11

**Authors:** Ho Hung Cheung, Wong Hoi She, Desmond Y.H. Yap, Simon H.Y. Tsang, Tan To Cheung

**Affiliations:** a Department of Surgery, Queen Mary Hospital, 102 Pok Fu Lam Road, Hong Kong, China; b Department of Medicine, University of Hong Kong, 102 Pok Fu Lam Road, Hong Kong, China; c Department of Surgery, University of Hong Kong, 102 Pok Fu Lam Road, Hong Kong, China.

**Keywords:** associating liver partition and portal vein ligation for staged hepatectomy, case report, hepatocellular carcinoma, liver resection, liver tumor, tumor lysis syndrome

## Abstract

**Rationale:**

Tumor lysis syndrome is a potentially lethal condition caused by rapid cell death, releasing a high level of toxic cytokines. It is common in patients with hematological malignancy but rare in solid tumors.

**Patient concerns:**

A 64-year-old patient presented to our unit with a 17.3-cm hepatocellular carcinoma and marginal liver reserve. The first-stage operation of associating liver partition and portal vein ligation for staged hepatectomy (ALPPS) was performed.

**Diagnosis:**

The patient was found to be anuric with grossly deranged electrolytes after the first-stage operation. Tumor lysis syndrome was diagnosed.

**Interventions:**

The patient was transferred to the intensive care unit for aggressive fluid administration and continuous venovenous hemofiltration for the management of tumor lysis syndrome.

**Outcomes:**

The patient recovered and then underwent the second-stage operation of ALPPS with extended right hepatectomy 8 days after the initial operation without any long-term sequelae.

**Lessons:**

ALPPS is a relatively new technique in liver surgery, entailing an increased risk of tumor lysis syndrome due to an in situ tumor after the first-stage operation. Clinicians should have a high index of suspicion regarding this potentially lethal complication with prompt management.

## 1 Introduction

Tumor lysis syndrome (TLS) is characterized by hyperuricemia, hyperkalemia, hypocalcemia, and hyperphosphatemia, released after extensive cell death from malignancy and overwhelmed the body's normal homeostasis.^[1]^ These electrolyte and metabolic disturbances can progress to clinical toxic effects, including renal insufficiency, cardiac arrhythmias, seizures, and mortality.^[2]^

TLS occurs when tumor cells release their contents into the bloodstream. While TLS is most frequently encountered after systemic treatment of aggressive hematologic malignancy, it is rare in solid tumors with only several case reports in the literature.^[3]^ The majority of TLS in solid tumors is associated with chemotherapy, while there is only a handful of TLS cases related to surgery.^[3]^ In this case report, tumor lysis syndrome occurred in a patient who had undergone initial surgery for a huge hepatocellular carcinoma (HCC).

## 2 Case report

Ethical approval was not necessary for the report as this was a retrospective review of anonymous clinical data. The anonymized patient has given permission to be included in the manuscript and has provided informed consent to the publication of the case.

A 64-year-old hepatitis-B virus carrier was referred to us for treatment of a massive HCC. Contrast triphasic computed tomographic scan showed a 17.3-cm tumor in the right liver lobe with arterial enhancement and portovenous washout (Barcelona Clinic Liver Cancer stage B).^[4]^ The alpha-fetoprotein level was 20 ng/mL. An indocyanine green retention test yielded a result of 20.0%, and the volume of the left lateral liver segment was 24% of the estimated standard liver volume. Liver enzyme levels were mildly elevated with a bilirubin level of 26 μmol/L, while the baseline renal function was normal. Given the patient's limited liver reserve, associating liver partition and portal vein ligation for staged hepatectomy (ALPPS) was scheduled.

The first stage of ALPPS took 3 hours and 18 minutes, with a blood loss of 500 mL. The moderately cirrhotic liver was occupied by a 17-cm tumor over the right lobe, compressing the right hepatic vein and the middle hepatic vein. Over 70% of the left medial segment was occupied by the tumor. Temporary clamping to mark the left liver lobe and an intraoperative indocyanine green retention test were performed. The overall time of clamping of the right hepatic artery was 15 minutes. The right portal vein was controlled with Hemolocks and divided. The liver was transected with Cavitron Ultrasonic Surgical Aspirator and Thunderbeat, with 2 20-minute cycles of Pringle maneuver (PM).

The patient developed anuria after the operation. Potassium rose to 5.4 mmol/L. Uric acid was elevated to 609 μmol/L while creatinine increased to 462 μmol/L. Phosphate went up to 2.52 mmol/L, and adjusted calcium dropped to 1.98 mmol/L. This fulfilled the Cairo and Bishop diagnosis of clinical TLS, which comprises at least 2 metabolic abnormalities, including hyperuricemia, hyperkalemia, hyperphosphatemia, and hypocalcemia, together with an increased creatinine level and anuria.^[1]^ The patient was transferred to the intensive care unit and was given generous intravenous isotonic fluid, frusemide, calcium chloride, and febuxostat. Continuous venovenous hemofiltration was started on postoperative day 1 and resulted in improved serum electrolytes. Urine output improved and was above 2.0 mL/kg/h since postoperatively day 2. Reassessment computed tomography showed patchy hypodense areas within the tumor and over the right liver parenchyma, suggestive of tumor and liver necrosis. The left lateral segment showed significant hypertrophy, amounting to 54% of the estimated standard liver volume. Hence, second-stage ALPPS with extended right hepatectomy was performed 8 days after the first operation. Histopathological examination reviewed a moderately differentiated HCC, measuring 17.0 × 15.0 × 11.5 cm, with lymphovascular permeation and portal venous invasion. The specimen had a clear resection margin, and multiple areas of necrosis were noted.

The patient's renal function subsequently improved, with a creatinine level similar to baseline. With further rehabilitation, the patient felt restored and returned home a few weeks after the second operation.

## 3 Discussion

Patients with significant tumor burdens and/or rapidly dividing tumors are at greatest risk for TLS.^[5]^ Other risk factors include chemosensitive tumors, a large area of tumor necrosis, and pretreatment renal dysfunction.

In the literature, there are only a handful of case reports of TLS in HCC, and the majority of them are related to transarterial chemoembolization for inoperable intrahepatic HCC^[6–8]^ or target therapy for metastatic HCC.^[9,10]^ Mehrzad et al^[11]^ reported a case of spontaneous TLS in metastatic HCC, and Lehner et al^[12]^ reported a case of TLS after radiofrequency ablation for HCC.

TLS in liver resection has never been reported, but the first case of TLS in ALPPS was encountered in less than a decade since the popularization of this treatment procedure.^[13]^ We postulate that the principles behind ALPPS lead to a higher risk of TLS. In any liver surgery, when major bleeding is encountered, PM is applied to limit the blood inflow in order to control the bleeding temporarily. Even though it has been shown that PM causes significant oxidative stress in human hepatocytes,^[14]^ in most cases, this will not cause any significant damage to the liver parenchyma, with the readily restored inflow after the release of the vascular clamp. However, since the portal vein to the diseased lobe is ligated in ALPPS, the liver may not recover from the stress caused by PM. This condition is further worsened by the presence of the in situ liver tumor after the first-stage operation. While HCC is more dependent on the hepatic artery supply, it can be damaged and undergo necrosis after PM. The intracellular content from dead tumor cells and the damaged portion of the liver is readily released into the systemic circulation before the second-stage operation, posing a constant threat of TLS. Moreover, most patients having ALPPS have a large tumor load, and thus compression and prolonged manipulation of the tumor during surgery are inevitable, further increasing the risk of TLS. Lastly, a heavy tumor burden with high cell turnover is commonly observed in these aggressive liver tumors, which makes up another essential predisposition for TLS.

TLS requires a high index of suspicion and prompt treatment due to its potential mortality, manifesting as renal failure, arrhythmia, and recurrent seizure. Compared with TLS in hematological malignancy, where the metabolic derangement occurs soon after the initial systemic treatment, TLS in solid tumors can have an insidious onset with a delay onset up to a few weeks.^[15]^ Due to its rare occurrence, difficulty in diagnosis, and subsequent treatment delay, the reported mortality of TLS in solid tumors is up to 35%, significantly higher than that in hematological malignancy at 1.9%.^[5]^

The principle of treatment covers 3 critical areas: hydration, correction of metabolic abnormalities, and treatment of renal failure. Aggressive fluid administration at 3 L per square meter per day reduces serum concentrations of harmful electrolytes, increases renal blood flow, and prevents crystal precipitation in renal tubules.^[2]^ To prevent uric acid crystals formation in renal tubules, uric-acid lowering agents such as allopurinol and febuxostat are also given to avoid renal failure. Compared with allopurinol, febuxostat has a higher potency due to its action to oxidized and reduced forms of xanthine oxidase.^[2]^ It also requires no renal adjustment and has a lower risk of hypersensitivity. In the case of renal failure, dialytic modality is needed. Continuous modality is preferred to intermittent hemodialysis to reduce the risk of “rebound” accumulation of toxic substances.^[16]^ In our patient, besides aggressive fluid resuscitation, febuxostat and hemofiltration were started once the diagnosis of TLS was established. He experienced no arrhythmia nor seizure and made a full recovery. This stresses the importance of early recognition and prompt treatment in patients with TLS, which is paramount to patients’ survival.

## 4 Conclusion

This is the first case report of TLS in ALPPS. Vigilance for this potentially lethal complication and prompt management may help prevent mortality and improve the safety of this novel surgical technique.

## Author contributions

**Conceptualization:** Wong Hoi She.

**Data curation:** Ho Hung Cheung, Wong Hoi She.

**Formal analysis:** Ho Hung Cheung.

**Investigation:** Ho Hung Cheung, Wong Hoi She, Desmond Yat Hin Yap, Simon Hing Yin Tsang.

**Methodology:** Ho Hung Cheung, Wong Hoi She.

**Project administration:** Ho Hung Cheung.

**Resources:** Tan To Cheung.

**Software:** Tan To Cheung.

**Supervision:** Wong Hoi She, Tan To Cheung.

**Validation:** Ho Hung Cheung, Desmond Yat Hin Yap, Simon Hing Yin Tsang.

**Visualization:** Wong Hoi She, Simon Hing Yin Tsang.

**Writing – original draft:** Ho Hung Cheung.

**Writing – review & editing:** Ho Hung Cheung, Wong Hoi She, Desmond Yat Hin Yap.
